# Low-Temperature Mechanical Properties of High-Density and Low-Density Polyethylene and Their Blends

**DOI:** 10.3390/polym13111821

**Published:** 2021-05-31

**Authors:** Ildar I. Salakhov, Nadim M. Shaidullin, Anatoly E. Chalykh, Mikhail A. Matsko, Alexey V. Shapagin, Ayrat Z. Batyrshin, Georgiy A. Shandryuk, Ilya E. Nifant’ev

**Affiliations:** 1 R&D Center PJSC Nizhnekamskneftekhim, Nizhnekamsk, Sobolekovskaya Str. 23, 423574 Republic of Tatarstan, Russia; shaidullinnm@mail.ru (N.M.S.); ajr-b@yandex.ru (A.Z.B.); 2 Frumkin Institute of Physical Chemistry and Electrochemistry, Russian Academy of Sciences (IPCE RAS), Leninskiy Prospekt 31, 119071 Moscow, Russia; chalykh@mail.ru (A.E.C.); shapagin@mail.ru (A.V.S.); 3 Boreskov Institute of Catalysis Siberian Branch of the Russian Academy of Sciences, Acad. Lavrentiev Prospect 5, 630090 Novosibirsk, Russia; matsko@catalysis.ru; 4 A.V. Topchiev Institute of Petrochemical Synthesis of Russian Academy of Sciences (TIPS RAS), Leninskii pr. 29, 119991 Moscow, Russia; gosha@ips.ac.ru (G.A.S.); ilnif@yahoo.com (I.E.N.)

**Keywords:** polyethylene, blends, low-temperature characteristics, mechanical properties, molecular structure

## Abstract

Low-temperature properties of high-density polyethylene (HDPE), low-density polyethylene (LDPE), linear low-density polyethylene (LLDPE), and their blends were studied. The analyzed low-temperature mechanical properties involve the deformation resistance and impact strength characteristics. HDPE is a bimodal ethylene/1-hexene copolymer; LDPE is a branched ethylene homopolymer containing short-chain branches of different length; LLDPE is a binary ethylene/1-butene copolymer and an ethylene/1-butene/1-hexene terpolymer. The samples of copolymers and their blends were studied by gel permeation chromatography (GPC), differential scanning calorimetry (DSC), ^13^C NMR spectroscopy, and dynamic mechanical analysis (DMA) using testing machines equipped with a cryochamber. It is proposed that such parameters as “relative elongation at break at −45 °C” and “Izod impact strength at −40 °C” are used instead of the ductile-to-brittle transition temperature to assess frost resistance properties because these parameters are more sensitive to deformation and impact at subzero temperatures for HDPE. LLDPE is shown to exhibit higher relative elongation at break at −45 °C and Izod impact strength at −20 ÷ 60 °C compared to those of LDPE. LLDPE terpolymer added to HDPE (at a content ≥ 25 wt.%) simultaneously increases flow properties and improves tensile properties of the blend at −45 °C. Changes in low-temperature properties as a function of molecular weight, MWD, crystallinity, and branch content were determined for HDPE, LLDPE, and their blends. The DMA data prove the resulting dependences. The reported findings allow one to understand and predict mechanical properties in the HDPE–LLDPE systems at subzero temperatures.

## 1. Introduction

High-density polyethylene (HDPE) is one of the most common engineering plastics produced on a large scale. Due to its good mechanical properties, rigidity, wear resistance, and chemical inertness, HDPE is widely applied in many areas of human activity.

HDPE can be produced as both a unimodal and a multimodal polymer [1]. These products are used for manufacturing plastic pipes, containers, bottles and films (i.e., for domestic consumption). However, in some specific industries (external coating of gas transportation pipelines), there is also a demand for HDPE, but stricter requirements are posed on its mechanical and impact resistance characteristics in this case [2,3]. There currently is interest in wider use of gas because the carbon footprint generated by its conversion is lower compared to that generated by consuming other renewable energy sources, which is an important aspect in terms of the environmental impact.

The low-temperature mechanical and impact properties of HDPE are extremely important for the external polyethylene coatings for steel pipelines and need to meet strict requirements. The reason for posing such strict requirements to frost resistance is that the pipes with insulation coatings are transported and laid at low temperatures (from −20 to −60 °C) in the regions with harsh climate such as Siberia and northern areas of Europe and America. Therefore, special focus is placed on maintaining the integrity of the polymeric coating on the pipes and improving the low-temperature mechanical properties of HDPE in order to ensure reliable and long-term performance of pipelines.

**Figure 1:**
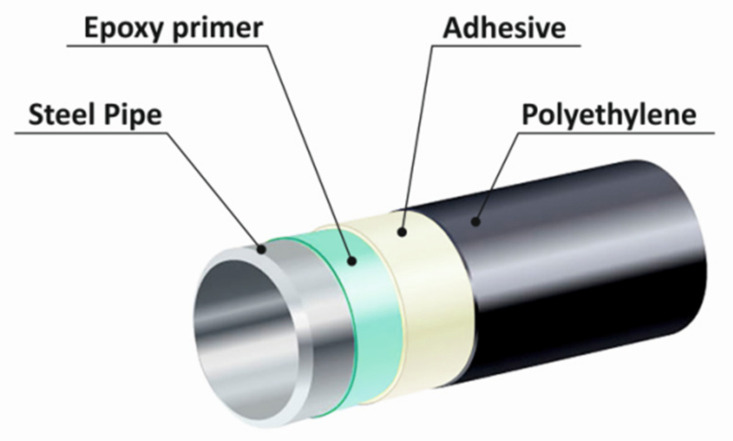
The composition of the three-layer insulation coating with the HDPE-based outer layer.

The systems consisting of two- or three-component layers (namely, polyethylene (most typically HDPE), an adhesive (most typically, the LLDPE-based one), and an epoxy primer) are used for manufacturing external coatings of steel pipes. The polyethylene layer is usually the thickest one in this system (3.0–3.5 mm), while the adhesive and primer layers are thinner (150–300 µm and 150 µm, respectively) (Figure 1) [4,5].

In this system, polyethylene prevents pipe exposure to the external environment, moisture, and oxygen; the primer protects it against corrosion, while the adhesive ensures the contact between PE and the primer [2]. The service life of such coatings shielding the pipes should be at least 20 years [5]; the preferred service life is as long as 50 years. According to the “Continental European coating” philosophy, mechanical impact resistance is the key parameter of the coating, which is ensured by the outer polyethylene layer in the three-layer system [6].

There are a number of national standards (DIN-30670, CAN/CAZ-Z245.21, and STO 2-2.3-130) [7,8,9] and the international standard ISO 21809-1 [10] posing requirements on the initial polyolefin materials and coatings based on them. However, not all the standards broadly regulate the low-temperature mechanical properties of polyethylene. Some of them only mention the requirements to impact strength (≥5 J/m) and ductile-to-brittle transition temperature (≤−70 °C). Polyethylene is characterized by good frost resistance due to its low ductile-to-brittle transition temperature (<−73 °C) [11]; however, this parameter cannot adequately show how the material behaves at subzero temperatures under real-world conditions. It should be emphasized that the ductile-to-brittle transition temperature value does not set the lower limit for the operating temperature of the end products; therefore, there exists a problem related to evaluation of frost resistance of PE. The ductile-to-brittle transition temperature only defines the temperature at which the brittle failure mechanism starts to be predominant, while the testing method does not take into account the time–temperature superposition principle [12]. Therefore, it is important to choose an adequate, informative, and suitable method for evaluating the behavior of PE-based materials at low temperatures as applied to the external coatings for steel pipes.

Quite a few publications have focused on frost resistance properties of polyethylene, but the key findings on this topic have been reported in refs [13,14,15,16,17,18,19,20,21,22,23,24]. A.N. Karasev et al. [23,24] studied the mechanical and impact strength properties of HDPE having different MWDs and showed that these properties are dependent on molecular weight and density. X. Lu et al. [17] investigated the LLDPE, MDPE, HDPE, and LDPE samples and demonstrated that their fracture toughness depends on molecular weight and density of linking molecules, while the yield strength rises with decreasing temperature due to the changes occurring in the noncrystalline region of polyethylene. K. Kitao [19] studied the low-temperature impact properties of LLDPE, MDPE, HDPE, and LDPE and revealed that impact strength and tensile strength tended to decline with decreasing test temperature. D. Weissmann and H. Alexander [12] focused on relative elongation at break for an LDPE film in the range between 0 and −60 °C at different strain rates and demonstrated that the “cold brittleness” temperature for the film stretched in the uniaxial direction was −52 °C. A.J. Peacock et al. [14] carried out the most thorough analysis of the effect of subzero temperatures on mechanical properties of linear and branched PE in the range between −100 and +140 °C.

The information about polyethylene used for steel pipe coatings is limited; the available studies focus on its properties at temperatures above zero. Few patents held by Borealis companies were related to the synthesis of reactor-grade polyethylene (e.g., refs. [25,26]), but a complex multiple-reactor scheme is used to produce such PE. Extrusion modification (including modification with LLDPE, LDPE, and other materials) is the simplest and most efficient method for fabricating insulating PE [3,27,28,29,30,31,32,33,34,35,36,37].

Hence, the small number of the currently available studies on frost resistance properties of HDPE (especially HDPE used for manufacturing pipe coatings) and the lack of systematic data on the effect of various factors on their properties necessitate the research in this field. The present study has both a fundamental and applied value because the gas sector is currently being developed and new alternatives for transportation of lighter gases (e.g., hydrogen) are being discussed. Furthermore, new hardly-accessible deposits in northern regions are discovered, so pipelines with a thermally stable polyethylene coating need to be laid. Therefore, research focused on polyethylene-based materials for manufacturing pipe coatings is rather relevant today.

This study aims at investigating the low-temperature mechanical properties of bimodal high-density PE and its blends with low-density PE. Determining the relationship between low-temperature mechanical properties and chemical structure of PE, thermal properties, and molecular characteristics will allow one to efficiently regulate the mechanical and impact strength properties of HDPE used to manufacture coatings for gas pipelines.

## 2. Experimental

### 2.1. Materials

Six samples of bimodal high-density polyethylene (ethylene/1-hexene copolymers) were the study objects. Their characteristics are summarized in Table 1.

The HDPE samples were synthesized via the two-reactor cascade scheme in gas-phase reactors in the bimodal regime over a titanium–magnesium Ziegler–Natta catalyst (TMC). The low-molecular-weight (LMW) fraction of high-density PE (>0.965 g/cm^3^) was synthesized in the first reactor by ethylene homopolymerization. The powder of ethylene homopolymer was then fed to the second reactor, where the low-density (<0.935 g/cm^3^) high-molecular-weight (HMW) fraction was synthesized on active sites of TMC due to ethylene/1-hexene copolymerization, and spherical particles consisting of a blend of the two polymers averaged at the extrusion stage by adding stabilizing agents were obtained. The 1-hexene content in the polymer and the LMW/HMW ratio in the bimodal HDPE were kept similar. The melt flow index of HDPE was varied by changing the amount of hydrogen fed into the second reactor.

Three samples of low-density polyethylene characterized by improved flowability and having short-chain (LLDPE) and long-chain (LDPE) branches were also studied. The characteristics of these samples are listed in Table 2. The LLDPE samples were also synthesized in the presence of titanium–magnesium catalyst. The LLDPE-2 sample was deliberately synthesized in gas-phase two-reactor systems. The synthesis involved ethylene/1-butene copolymerization in both reactors, and 1-hexene was added as the third comonomer. In other words, a ternary copolymer (ter-co-LLDPE) modified with 1-hexene was obtained. Furthermore, the molecular weight distribution (MWD) of this ter-co-LLDPE was slightly modified (broadened) by varying the amount of hydrogen fed into both reactors.

The compositions (Table 3) based on HDPE (sample 4) and LLDPE (sample 2) were obtained using a TSE24MC double-screw extruder (Thermo Fisher Scientific) with screw diameter (D) = 24 mm and the L/D ratio = 40. Temperature in the extruder zones was 245–250 °C. A mixture of phenolic antioxidant and phosphite heat stabilizer (0.15 wt.% each) was added for stabilization.

### 2.2. Methods

The density of the polyethylene samples was measured according to the ASTM D1505-3 standard test method using a density gradient column. The melt flow index was analyzed at 190 °C under loads of 5 kg in accordance with the ASTM D 1238 standard test method.

The molecular weight characteristics of the polyethylene samples were analyzed by gel permeation chromatography on a PL-220 instrument equipped with a refractometric sensor and a differential viscometer. 1,2,4-Trichlorobenzene was used as a solvent (test temperature, 160 °C; flow rate, 1 cm^3^/min). The calibration curve was plotted using polyethylene and polystyrene reference standards in a broad range of molecular weights (from 540 to 13,200,000). The data were analyzed using the conventional or universal calibration based on the relationship determined in ref. [33]. According to it, the following equation is true for two polymers characterized by the same retention time (t):

(*M*_st_ × [*η*]_st_)_t_ = (*M*_x_ × [*η*]_x_)_t_, (1)

where *M*_st_ is the molecular weight and [*η*]_st_ is the characteristic viscosity of the known reference standard; *M*_x_ is the molecular weight and [*η*]_x_ is the characteristic viscosity of the polymer under study.

The polymers were fractionated on a PolymerChar PREP mc^2^ automated setup (Spain). LLDPE-1 and LLDPE-2 were separated into fractions with narrow MWD using the following procedure: 1 g of the sample was dissolved in 200 mL of xylene for 2 h; a calculated amount of 2-(2-butoxyethoxy)ethanol was then added to the polymer solution for precipitating some of the polymer. The hot polymer solution was filtered off into a receiving flask. The precipitate was dissolved in a new portion of xylene, and the precipitation/dissolution procedure was continued until five fractions with *M*_w_/*M*_n_ ≤ 2 were obtained. The LLDPE-1,2 samples were separated into five fractions (F1–F5) at the volume concentration of 2-(2-butoxyethoxy)ethanol of 60, 50, 45, 41, and 0 vol.%. The fractions were precipitated with acetone; the polymer precipitate was filtered off from the mother liquor and dried to constant weight.

The number of CH_3_ groups in HDPE, LLDPE, and polyethylene-based compositions was determined by IR spectroscopy on an FTIR-8400S Fourier Transform Infrared spectrometer (Shimadzu) (the number of scans, 50; resolution, 2.0). The samples were prepared as 150–250-µm thick films. The number of CH_3_ groups was determined according to the band at 1378 cm^−1^ corresponding to bending vibrations of methyl groups using the procedure described in refs [34,35].

The number of CH_3_ groups per 1000 C atoms was determined by NMR. The ^13^C NMR spectra were recorded in standard 10 mm cylindrical ampoules on a Bruker MSL-400 spectrometer at a frequency of 100.612 MHz at 99 °C. The accuracy and reproducibility of temperature setting was 1 °C. Scan parameters were as follows: sweep rate, 30 kHz; scan rate, 0.1 Hz; total number of scans, 1000–10,000. The chemical shifts of the signals in the ^13^C NMR spectra were determined with respect to the internal standard (the signal from carbon atoms in the C–Cl fragments of an o-DFB with chemical shift of 132.4 ppm).

The thermal characteristics of the samples were studied by differential scanning calorimetry on a DSC-204F1 Phoenix instrument (Netzsch) in the dynamic mode of heating/cooling in an argon atmosphere at a heating rate of 10 °C/min from 50 to 210 C. The crystallinity of the test samples was determined using this formula:

(2) $$\chi =\frac{\Delta H_{\mathrm{fus}}}{\Delta H_{\mathrm{fus}\;100\%}}\cdot 100\%$$

where $\Delta H_{\mathrm{fus}}$ is the enthalpy of fusion of the sample and $\Delta H_{\mathrm{fus}\;100\%}$ is the enthalpy of fusion of the 100% crystalline sample (293 J).

The ductile-to-brittle transition temperature was determined in accordance with the state standard GOST 16783 (method B). According to this method, the sample folded so as to form a loop was subjected to deformation at test temperature of −70 °C, followed by determining the fracture or cracks visible to the unaided eye. The samples 0.5 mm thick, 40 mm long, and 6 mm wide were secured in holders, cooled down to the given temperature, exposed to it for 2 min, and then deformed at a rate of 2 m/s. It was considered that the tests have been passed if none of the five tested samples developed any defects.

Sheets 2 and 3 mm thick were pressed for conducting the deformation resistance tests under uniaxial extension and impact strength tests, respectively. The pressing temperature was 180 °C; pressure was 8 N/cm^2^; time was selected with allowance for sample thickness (+5 min per each mm of the sample). The cooling rate was 20 °C, and the pressing end temperature was 40 °C. Samples for the tensile test and notched Izod impact test were punched out of the sheets in accordance with the ASTM D882 and ASTM D256 standard test methods, respectively.

The mechanical properties of PE and PE-based compositions at +23 °C were determined in accordance with the ASTM D882 standard test method at a clamp holder spreading rate of 100 and 300 mm/min. The deformation resistance properties at −45 °C were measured on an AI-7000S setup (Gotech) in accordance with the ASTM D882 standard test method at a clamp holder spreading rate of 50 mm/min. The samples were exposed to the predetermined temperature for 30 min.

The impact characteristics of the compositions were evaluated using the “Izod impact strength” parameter in accordance with the ASTM D256 standard test method. The notch cut was made on a milling machine. The tests were conducted at 23 °C and at subzero temperatures (from 0 to −100 °C with an increment of 20 °C). A mixture of carbonic acid gas and ethanol (at test temperatures ranging from 0 to −60 °C) and a mixture of liquid nitrogen and 1-hexene (at test temperatures ranging from −80 to −100 °C) were used as cooling agents. The ratios between the components of the cooling mixture were determined depending on the test temperature. The samples were subjected to abrupt cooling and exposed to this temperature for 1 h; the tests were then performed for a short time. The nil ductility temperature was determined graphically from the Izod impact strength data according to the procedure described in ref [19].

Dynamic mechanical analysis (DMA) was conducted on a DMA1 instrument (Mettler Toledo, Switzerland). The tests were performed in the dynamic heating mode in the temperature range from −150 to +100 °C at a heating rate of 3 K/min. The measurements were performed in the dual cantilever mode. The loading amplitude was up to 10 N; the deformation amplitude was 50 µm; the frequency was 1 Hz. The typical sample size was as follows: length L = 20 mm; width W = 3 mm; thickness T = 2 mm; and the geometric factor G (G =L^3^/16WT^3^) was 2.0833 × 10^4^ m^−1^.

## 3. Results and Discussion

In the modern approach to manufacturing plastic materials, there is a trend toward reducing the processing cycle time and thickness of the end products, while retaining their high-performance characteristics. Therefore, the field of applying coatings onto steel pipes has also evolved since the late 20th century. Thus, whereas circumferential coating deposition using PE with low flow properties (in particular, carbon black filled LDPE) was previously employed during extrusion, the modern method involves flat die extrusion of high-flow carbon black filled materials based on bimodal HDPE [25,26]. The key differences for producing modern pipelines are as follows: high coating application rate; the use of HDPE with enhanced melt flow behavior (MFI5.0 = 1.5 ÷ 2.5 g/10 min or MFI2.16 = 0.35 ÷ 0.6 g/10 min) [11], and small thickness of the resulting PE coating (≤2 mm). Therefore, this specific processing field requires composite materials characterized by a combination of improved rheological characteristics (high flowability) and mechanical properties, as well as high resistance of the polyethylene-based coating to subzero temperatures.

At the first stage of this study, we investigated a series of bimodal HDPE samples; LDPE and LLDPE were studied at the second stage; and the HDPE/LLDPE blends, at the third stage.

### 3.1. Molecular and Thermal Characteristics and Low-Temperature Properties of Bimodal HDPE

Bimodal HDPE samples having similar density and crystallinity but different molecular weight (*M*_w_). MWD and MFI were deliberately synthesized to study the low-temperature deformation resistance and impact strength properties, as well as ductile-to-brittle transition temperature (Table 4).

Table 4 lists the results of studying the HDPE samples.

Table 4 shows that increasing hydrogen concentration in the second reactor (where the ethylene/1-hexene copolymer is formed) during the synthesis of bimodal HDPE results in reduction of the weight average molecular weight (*M*_w_) and narrowing of the molecular weight distribution (*M*_w_/*M*_n_) of the polymer. Height of the MWD peak of bimodal HDPE corresponding to the high-molecular-weight (HMW) fraction decreases, which is also confirmed by a decline in the sedimentation average molecular weight (*M*_z_). Thus, whereas *M*_w_ and *M*_z_ for HDPE-1 are equal to 250 and 980 kg/mol, respectively, these parameters decrease for HDPE-4 to 160 and 720 kg/mol, respectively. The melt flow index MFI_5.0_ increases fivefold in this case (from 0.2 to 1.0 g/10 min), while increasing tenfold (from 0.2 to 2.0 g/10 min) for the HDPE-6 sample. The trend towards changes in crystallinity can be attributed to variation of density. The relationship between these parameters is linear and depends on comonomer content and distribution within polyethylene (the melting point is not changed considerably).

Variation of the HDPE synthesis conditions within the studied range of molecular weight and melt flow index (regardless of the tenfold change in the MFI) had no significant effect on the ductile-to-brittle transition temperature; it was less than −70 °C for all the samples (Table 4). As mentioned above, due to the low ductile-to-brittleness temperature (*T*_db_), PE is considered an impact-resistant polymer at subzero temperatures. It is not quite true because the *T*_db_ values usually found in reference books typically refer to static tests, while *T*_db_ increases abruptly under loading (the pattern of its increase may vary depending on the supramolecular structure.) In our opinion, *T*_db_ cannot unambiguously represent the behavior of a material at subzero temperatures and cannot set the lower limit for the operating temperature of the materials being used. Therefore, in order to more reliably assess the frost resistance of HDPE, we suggest investigating the deformation resistance and impact strength properties at subzero temperatures; namely, such parameters as relative elongation at break at −45 °C (Ɛ_b_^−45°C^) and Izod impact strength at −40 °C (A_Izod_^−40°C^) were selected.

**Figure 2:**
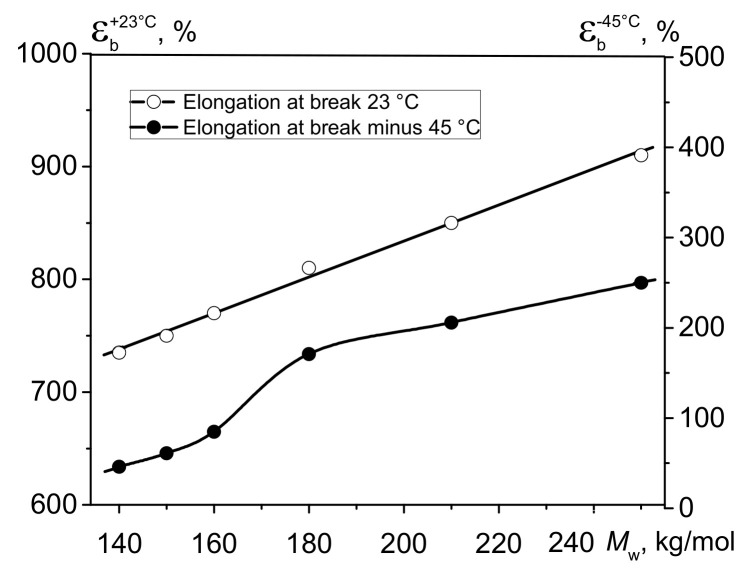
Relative elongation at break at test temperatures of +23 °C (Ɛ_b_^+23^) and −45 °C (Ɛ_b_^−45^) as a function of *M*_w_ for the bimodal HDPE samples.

**Figure 3:**
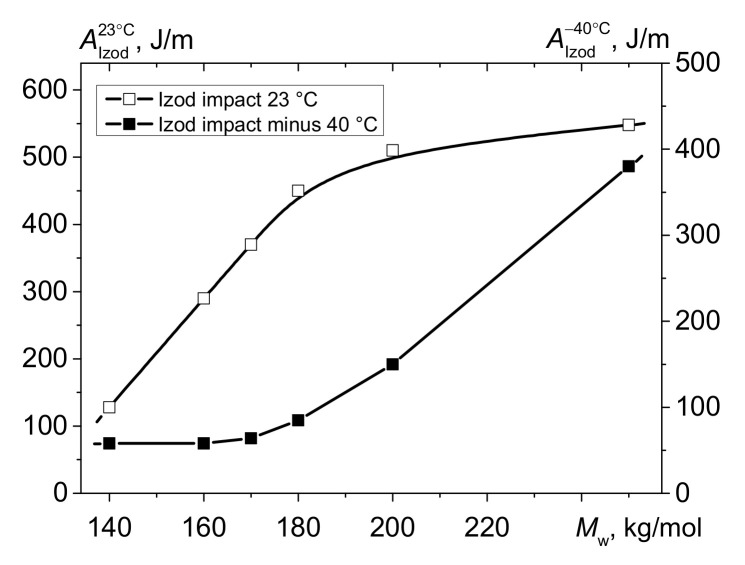
Izod impact strength at test temperatures of +23 °C (A_Izod_^+23^**)** and −45 °C (A_Izod_^−40^) as a function of *M*_w_ for the bimodal HDPE samples.

The curves showing the dependence of relative elongation at break at −45 °C and Izod impact strength at −40 °C on *M*_w_ were plotted for the bimodal HDPE samples (Figure 2 and Figure 3). The dependences of these parameters for HDPE recorded at +23 °C are also presented.

Overall, it has been determined that the relative elongation at break at test temperatures of +23 and −45 °C for HDPE declines with decreasing molecular weight (Figure 3), but the curve shapes are different. The curve showing Ɛ_b_^+23°C^ as a function of molecular weight is linear and descends proportionally from 900 to 735%. Meanwhile, the curve showing the dependence of Ɛ_b_^−45°C^ on *M*_w_ is S-shaped and descends abruptly to <100% in the *M*_w_ range of 150 ÷ 200 kg/mol (MFI_5.0_~0.5 ÷ 1.5). Thus, whereas the relative elongation at break at −45 °C for HDPE-1 is 250% (all other conditions being identical), it decreases to 85% and 46% for HDPE-4 and HDPE-6, respectively. In other words, Ɛ_b_^−45°C^ drops~5.5-fold as *M*_w_ decreases almost twofold (from 250 to 140 kg/mol)

The curves showing the dependence of the Izod impact strength at +23 °C and −40 °C on *M*_w_ for bimodal HDPE also descend with decreasing molecular weight of the polymer, while the shapes of the curves differ. Unlike the A_Izod_^-+23°C^ *M*_w_ curve, the A_Izod_^−40°C^ *M*_w_ curve is downsloping (Figure 3). Thus, whereas the Izod impact strength for HDPE-1 is 380 J/m, it decreases to almost the same level (64 and 58 J/m (i.e., almost sixfold)) for HDPE-4 and HDPE-6, respectively.

Hence, the observed changes in the mechanical and impact strength properties of bimodal HDPE at subzero temperatures were found to correlate with reduction of molecular weight, the content of HMW fraction containing branches, and narrowing of the MWD of HDPE, which may be attributed to two cumulative reasons. First, the probability that several crystals contain one macromolecule (when the same macromolecule can act as a crystal seed for several crystals) decreases as macromolecule length declines. Second, the number of long macromolecules enriched in branches that were formed due to comonomer addition decreases at lower contents of the high-molecular-weight fraction. These changes have an unfavorable effect on low-temperature mechanical properties because the branched high-molecular-weight fraction of the polymer enables redistribution of stresses emerging in the material and reduces the probability of sample failure caused by crack propagation at the instant when it is deformed [15,17].

**Figure 4:**
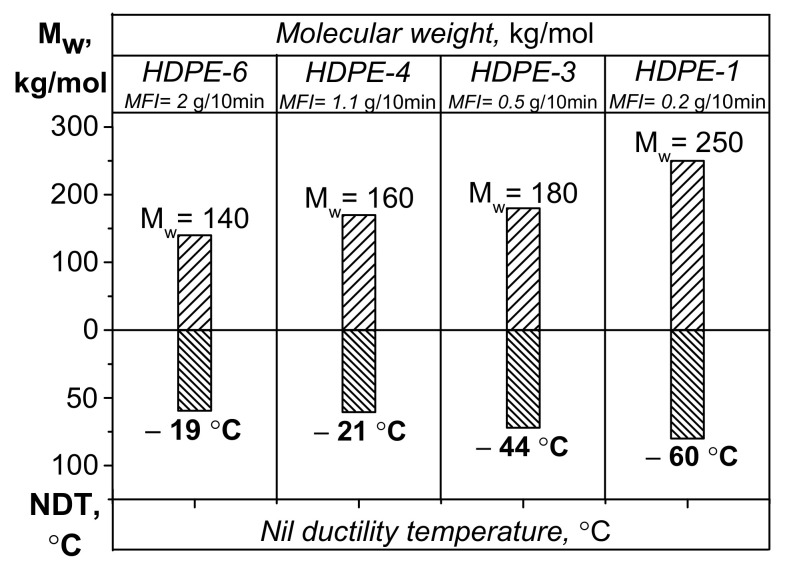
The diagram showing the dependence between the calculated parameter “nil ductility temperature (NDT)” [19] and *M*_w_ for the bimodal HDPE samples.

The aforementioned findings were also confirmed by the nil ductility temperature (NDT) parameter, which was proposed by K. Kitao [19] to indicate the ductile-to-brittle transition for polyethylene (Figure 4). The nil ductility temperature is determined by calculations (graphically) according to impact strength values (in this study, within the temperature range from +23 °C to −60 °C). This parameter reveals the “critical” temperature at which PE becomes embrittled and the probability of formation of defects and cracks contributing to polymer failure at subzero temperatures increases.

Figure 4 shows that the nil ductility temperature correlates well with changes in molecular weight of the HDPE samples and shows the instant when the material becomes brittle, resulting in higher probability of crack formation during exposure to deformation and impact loading. A conclusion can be drawn from the reported data that elasticity is significantly reduced at low temperatures for the HDPE samples with *M*_w_ < 180 kg/mol (and MFI > 0.5 g/10 min). Such behavior of PE is presumably caused by reduction of mobility in the noncrystalline phase and content of the high-molecular-weight fraction with branched macromolecules.

Hence, the results of the studies of bimodal HDPE samples having similar density and crystallinity values but different *M*_w_ and MWD showed that low-temperature properties of PE worsen with decreasing molecular weight as the polymer elasticity decreases and its brittleness increases at subzero temperatures. It is clear that such parameters as relative elongation at break at −45 °C and the Izod impact strength at −40 °C for HDPE more completely characterize the low-temperature stability of PE compared to the “ductile-to-brittle transition temperature” parameter.

In order to improve properties of HDPE, we have chosen the method for melt modification of the polymer yielding a composition characterized by high machinability and resistance to low temperatures. Therefore, the next stages of our study involved searching for and studying the modifying agents of close nature that would allow one to simultaneously enhance the melt flow characteristics and improve the low-temperature tensile and impact properties of the composition.

### 3.2. Low-Temperature Properties of LLDPE and LDPE

An efficient method for improving the mechanical and rheological properties of HDPE is to modify it by blending with low-density polyethylene either containing short-chain branches (LLDPE) or simultaneously containing short- and long-chain branches (LDPE) [36,37]. The binary copolymers with a single comonomer (1-butene, 1-hexene, or octene-1) produced over Ziegler–Natta catalysts have been well-studied in the available literature. However, there are very few publications focused on copolymers with two α-olefins and a limited number of studies focused on their low-temperature properties. When studying the variants of HDPE modification, we selected three low-density materials characterized by enhanced (and similar) melt flow index (~6–8 g/10 min) and differing in their molecular structure: LDPE produced by free-radical polymerization and containing short- and long-chain branches, the binary ethylene/1-butene copolymer (LLDPE-1(bi-co-LLDPE)), and ethylene/1-butene/1-hexene terpolymer LLDPE-2 (ter-co-LLDPE) (Table 2).

One can see that PE samples differ in terms of their micro and macrostructure. LDPE is characterized by a broad combination of short-chain branches of different length, the presence of long-chain branching and a broad MWD, but the content of fractions soluble in o-xylene is low (5.5 wt.%). The ethylene/1-butene copolymer (EBC) has a narrow MWD and an elevated comonomer content; therefore, the content of the amorphous fraction is increased. The deliberately synthesized ethylene/1-butene/1-hexene terpolymer (see the Section 2) is also characterized by the unimodal shape of MWD curves, but has a broader MWD (*M*_w_/*M*_n_ = 5.2) compared to that of the ethylene/1-butene copolymer (*M*_w_/*M*_n_ = 3.2). The LLDPE-2 terpolymer has a higher content of the fraction soluble in o-xylene (14.0 wt.%) compared to that in the binary LLDPE-1 copolymer (9.5 wt.%).

The LLDPE fraction soluble in o-xylene can be named “notionally amorphous” (because of the reduced molecular weight) because it is similar to polyolefin elastomers in terms of its phase state. This fraction contains a large number of comonomer units. According to the DSC data, its glass transition temperature corresponds to −51 °C; therefore, this fraction can be classified as very low density or ultra-low-density polyethylene that typically has glass-transition signals in this temperature range [38].

**Figure 5:**
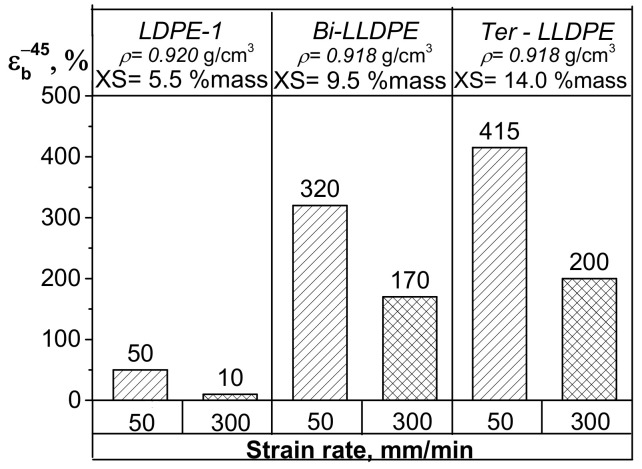
The data on ε^−45^ for the LDPE, LLDPE-1, and LLDPE-2 samples at different strain rates (50 and 300 mm/min).

**Figure 6:**
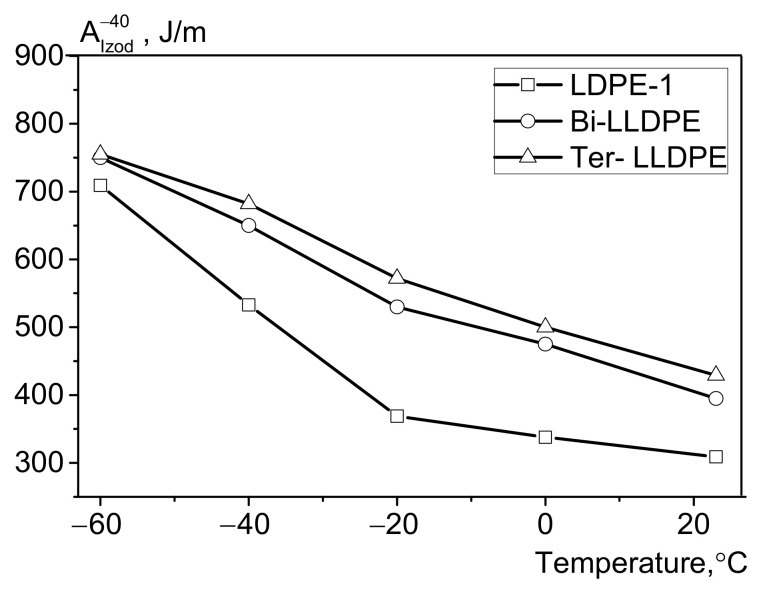
The data on impact strength for the LDPE, LLDPE-1, and LLDPE-2 samples in the temperature range from −60 °C to +23 °C.

Investigation of the low-temperature properties of the LDPE and LLDPE samples revealed significant differences in their mechanical characteristics at subzero temperatures (Figure 5). LDPE was found to have a low relative elongation at break at −45 °C (50%) compared to that of LLDPE (Ɛ_b_^−45°C^ > 300%). Interestingly, a difference between the LLDPE-1 and LLDPE-2 copolymers was also revealed. Thus, whereas the relative elongation at break at −45 °C for binary ethylene/1-butene copolymer (provided that all other test conditions are identical) is 323%, Ɛ_b_^−45°C^ for the 1-ethylene/butene/1-hexene tercopolymer is 415% (i.e., ~20% higher). The increase in the strain rate from 50 to 300 mm/min during tensile tests also proved that Ɛ_b_^−45^ differed for bi-co-LLDPE-1 (172%) and ter-co-LLDPE-2 (202%). Investigation of impact strength characteristics revealed that the Izod impact strengths at −40 °C (A_Izod_^−40°C^) also differ for LDPE and LLDPE (Figure 6). The ethylene/1-butene/1-hexene copolymer was shown to have the highest impact strength. As for the trend towards variation of shock viscosity as a function of temperature, these changes are seemingly related to the gradual increase in the Young’s modulus of LDPE (LLDPE) with decreasing temperature and reduction of the probability of sample bending when exposed to impact loading. This behavior of polymers characterized by low Young’s modulus is not a new phenomenon and has been reported in the available literature [39].

**Figure 7:**
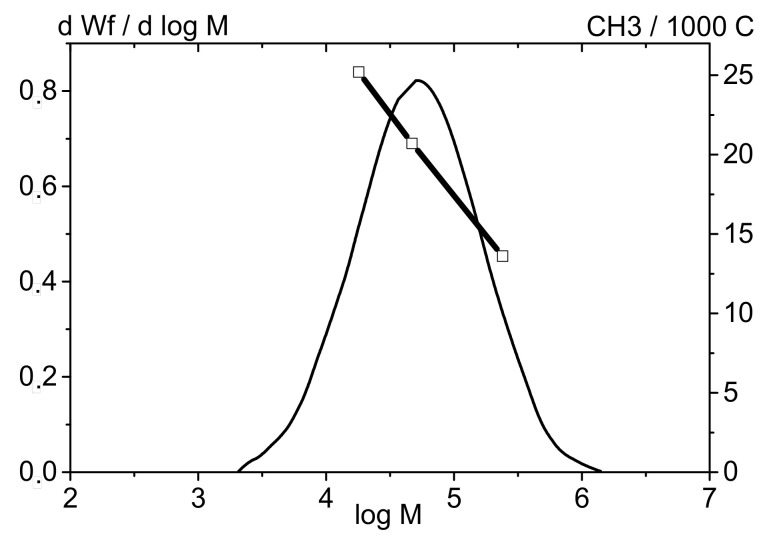
The curves showing molecular weight distribution (1) and content of the comonomer (1-butene) in LLDPE-1 fractions.

**Figure 8:**
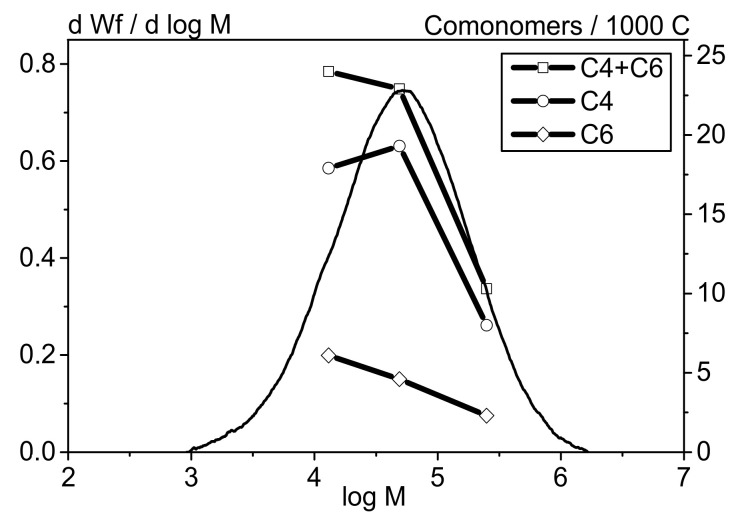
The curves showing molecular weight distribution (1), total content of the comonomers (2), content of the comonomer (1-butene) (3), and content of the comonomer (1-hexene) (4) in all the LLDPE-2 fractions.

It was shown by comparing the bi-co-LLDPE-1 and ter-co-LLDPE-2 samples that the type of comonomer and its distribution in PE macromolecules has an effect on frost resistance properties of LLDPE provided that all other conditions (MFI, MWD, total comonomer content and density) are identical (Table 5, Figure 7 and Figure 8)).

Studying the structural features of the bi-co-LLDPE-1 and ter-co-LLDPE-2 samples using the fractionation method followed by an analysis of the isolated fractions by IR spectroscopy and NMR and plotting the branching distribution profile depending on molecular weight revealed that the comonomer distribution in the MWD of LLDPE has a shape typical of that for the polymers produced over Ziegler–Natta catalysts. For these polymers, the high content of comonomer units is observed in the low-molecular-weight region and, vice versa, the content of α-olefin units in the high-molecular-weight region is low [40,41,42,43,44].

The total content of branches (butene and hexene monomers for ter-co-LLDPE-2; butene monomers for bi-co-LLDPE-1) was found to be almost identical in the low-molecular-weight region of MWD for both copolymers (LLDPE-1,2). However, it was revealed by comparing these LLDPE samples that while they are characterized by appreciably similar comonomer contents (~21–22 CH_3_/1000C), there are some differences in the compositional heterogeneity of the copolymers under study. It was found that the content of α-olefin monomers within the medium-molecular-weight range (log4–5/50–100 × 10^3^) is higher for the terpolymer compared to that for the binary polymer. The content of comonomer units is increased because butene monomers are predominating. The curve showing the distribution of hexene monomers in LLDPE-2 is smoother and more uniform than that for butene monomers, for which there typically is a peak at lg4.7. Meanwhile, the content of butene monomers within the high-molecular-weight fraction region is higher for bi-co-LLDPE-1 compared to that for ter-co-LLDPE-2. As mentioned above, these differences are caused by the selected conditions of synthesizing LLDPE terpolymers and a broader MWD. The improved Ɛ_b_^−45°C^ and A_Izod_^−40°C^ values for ter-co-LLDPE-2 are presumably caused by the difference in MWD, addition of 1-hexene, and distribution of the comonomers. Overall, 1-hexene reduces density and crystallinity more efficiently compared to 1-butene; therefore, it significantly modifies the amorphous region characterized by limited mobility (the so-called “rigid–amorphous fractions” [44,45,46]), which is the intermediate region between the crystalline and amorphous fractions. These changes have a favorable effect on low-temperature properties of LLDPE.

Hence, the results of the tests showed that ethylene/1-butene/1-hexene terpolymer exhibits the best low-temperature properties (ter-co-LLDPE > bi-co-LLDPE > homo-LDPE). The revealed advantages are caused by the features of the synthesis of ter-co-LLDPE-2 via the two-reactor scheme (namely, by a combination of two types of comonomers, their distribution, branching degree, and the molecular weight structure). The findings made it possible to choose the optimal type of LLDPE for modifying the bimodal HDPE characterized by enhanced flow properties.

### 3.3. Binary Compositions Based on HDPE and LLDPE

The next stage involved modifying the bimodal HDPE (ethylene/1-hexene copolymer) via extrusion melt compounding with ter-co-LLDPE-2 (the ethylene/1-butene/1-hexene terpolymer) at different ratios (the blend ratios are shown in Table 3).

**Figure 9:**
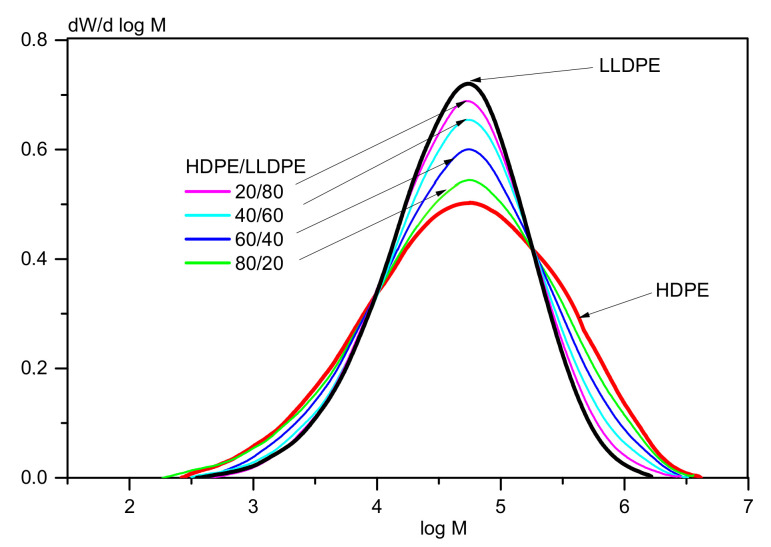
The MWD curves of the samples of bimodal HDPE and ter-co-LLDPE and their blends.

Table 6 demonstrates that adding LLDPE to HDPE expectedly increases the MFI and reduces *M*_w_, while the MWD becomes narrower. The MWD curves for HDPE and LLDPE are characterized by bimodal and unimodal distribution, respectively (Figure 9).

The shape of the MWD curves for binary blends depends on the HDPE/LLDPE ratio in them. The peak on the MWD curve of the bimodal HDPE referring to the high-molecular-weight fraction becomes lower and is almost not detected as the LLDPE content is increased to ≥40%. The density and crystallinity of the blends also decreases proportionally with rising LLDPE content.

**Figure 10:**
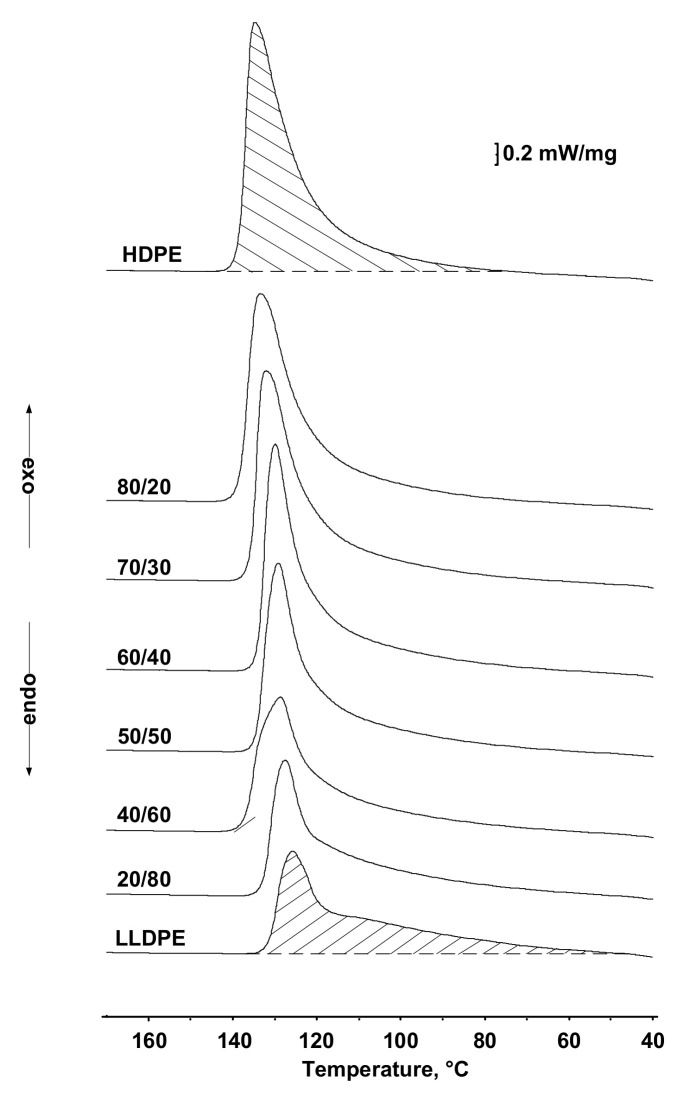
The effect of LLDPE on the DSC curves of HDPE/LLDPE blends.

Figure 10 shows the typical exothermal melting curves for all the polyolefin samples and their blends. One can see that each exothermic curve has only one melting and crystallization peak whose position depends on composition of the blend. The melting point was gradually shifted from 135 to 126 °C with increasing LLDPE content.

Investigation of mechanical properties showed that adding LLDPE to HDPE increases the relative elongation at break for the HDPE/LLDPE blends at test temperatures of both +23 °C and −45 °C. However, the shapes of the curves differ, which starts to be detectable at LLDPE contents in HDPE > 20 wt.%. Whereas the relative elongation after adding LLDPE increases linearly at test temperature of +23 °C, the curve showing the relative elongation at −45 °C as a function of LLDPE content is S-shaped.

**Figure 11:**
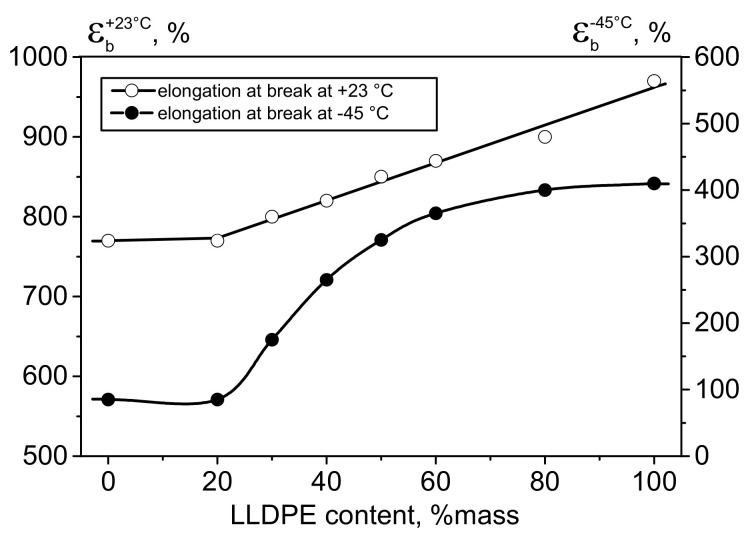
The effect of weight fraction of LLDPE in HDPE on relative elongation at break at +23 and −45 °C.

In the case of testing deformation resistance properties at subzero temperatures for the initial HDPE, the Ɛ_b_^−45°C^ value was 85%, being equal to 180% (i.e., twice as high) for the blend with HDPE/LLDPE = 70/30 (Figure 11). The rise in relative elongation at break at −45 °C for the blends seems to be caused by the increasing content of comonomer units (i.e., increasing degree of branching) (Table 6). The content of the amorphous fraction increases, while crystallinity and ductility decrease [34], thus exhibiting a favorable effect on elasticity at low temperatures.

**Figure 12:**
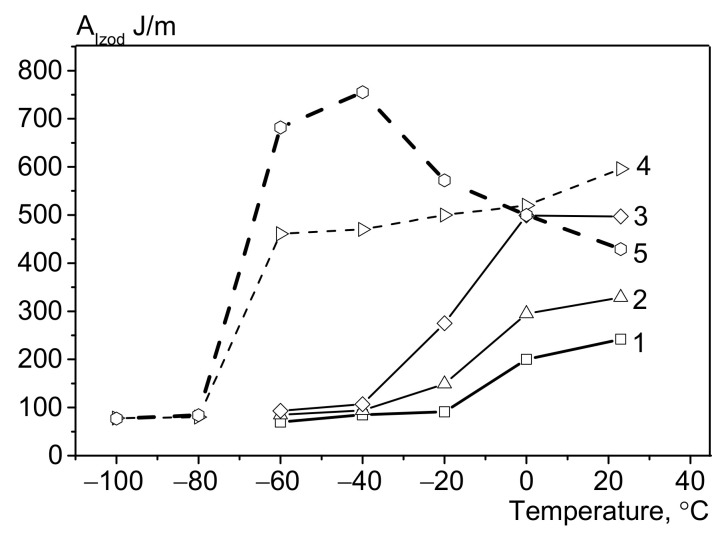
The Izod impact strength of HDPE, LLDPE, and their blends at different test temperatures: HDPE (1), HDPE/LLDPE = 70/30 (2), 50/50 (3), 20/80 (4), and LLDPE (5).

The Izod impact strength tests of the samples revealed that impact strength of LLDPE is higher than that of HDPE, and the difference between these values depends inversely on the test temperature (Figure 3, Figure 6 and Figure 12). Figure 12 shows that the Izod impact strength of bimodal HDPE decreases at subzero temperatures, while remaining virtually unchanged (80–95 J/m) within the range from −20 to −60 °C. Contrariwise, the impact strength of LLDPE is high in a broad range of subzero temperatures (between −20 and −60 °C). This can be attributed to the fact that LLDPE is a ductile material; therefore, by the end of the exposure to impact loading, multiple microvoids are formed at the end of the notch, and the sample is stretched around them. These microvoids make it possible to evenly distribute the emerging stresses and reduce the probability of further fatigue crack extension. This concept of the deformation mechanism under impact loading is not new and is known as “crack shielding” [14]. Therefore, the rising content of LLDPE in the binary blends increases the probability of crack shielding by increasing the impact strength values at a given temperature. Blending HDPE with LLDPE allows one to significantly (severalfold) increase the Izod impact strength at +23 °C for the HDPE/LLDPE blends. However, in the low-temperature range, the impact strengths did not differ significantly from those of the initial HDPE at temperatures from −40 to −60 °C regardless of the HDPE/LLDPE ratio (80/20–20/80); in other words, addition of LLDPE has no effect on impact strength of blend compositions.

**Figure 13:**
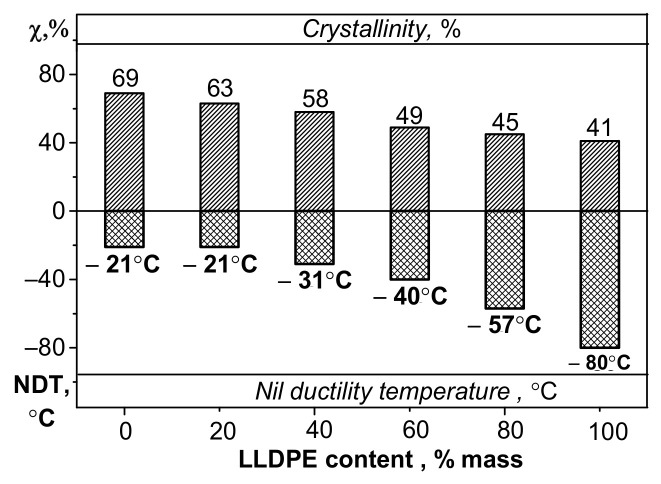
The effect of weight fraction of LLDPE on nil ductility temperature.

The nil ductility temperature of the blends decreases with rising LLDPE content (Figure 13), which is related to the high low-temperature properties of LLDPE. However, no correlation between the nil ductility temperature and impact strength was revealed within the temperature range between −60 and −40 °C.

**Figure 14:**
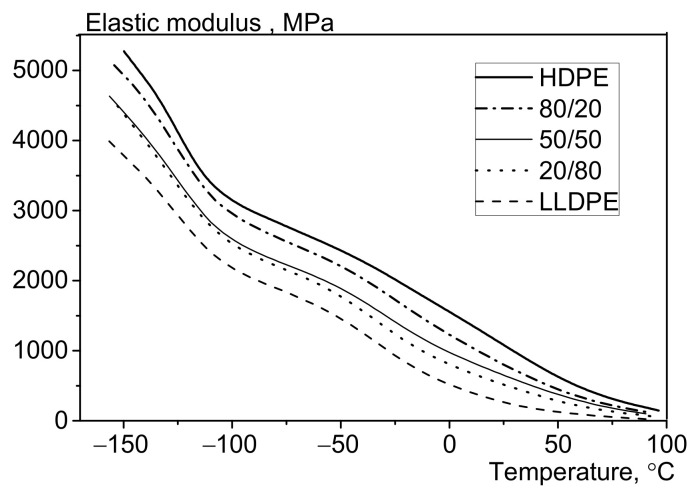
The effect of LLDPE on the elasticity modulus of the HDPE/LLDPE blends (DMA).

**Figure 15:**
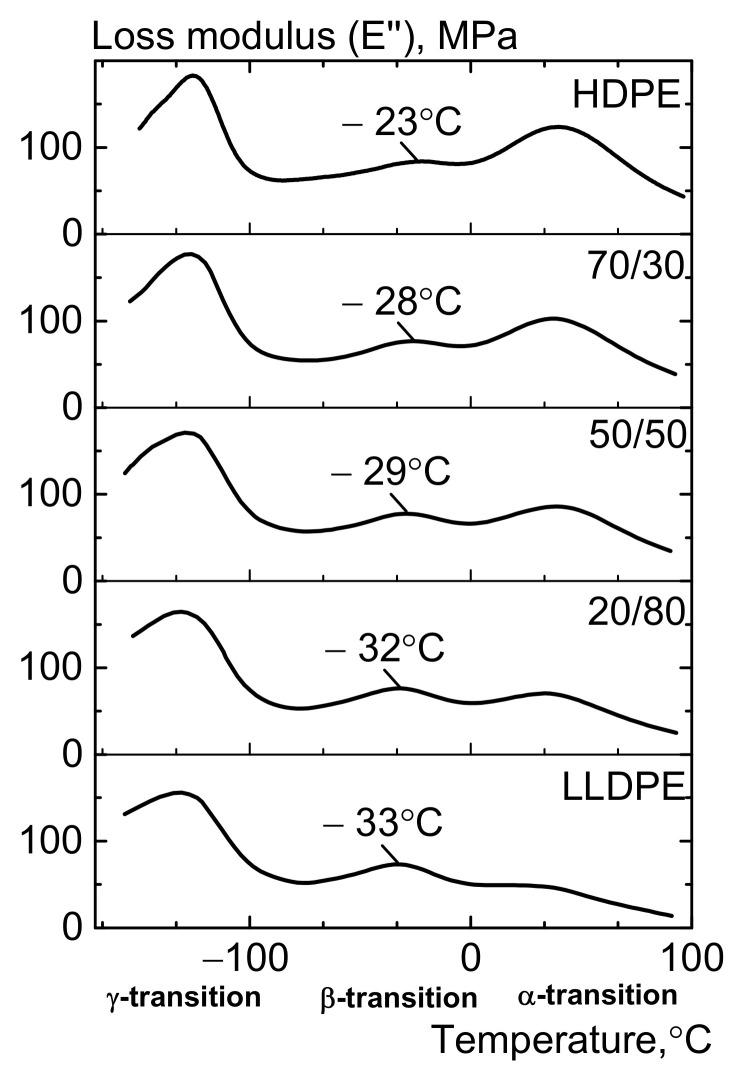
The effect of LLDPE on the loss modulus of the HDPE/LLDPE blends (DMA).

**Figure 16:**
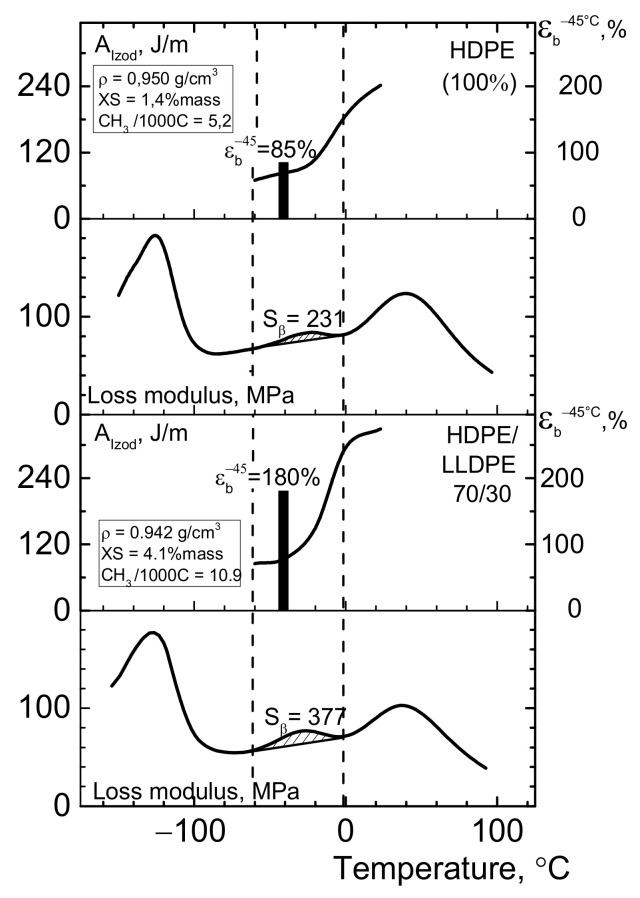
The results of DMA of HDPE, LLDPE, and their blends.

**Figure 17:**
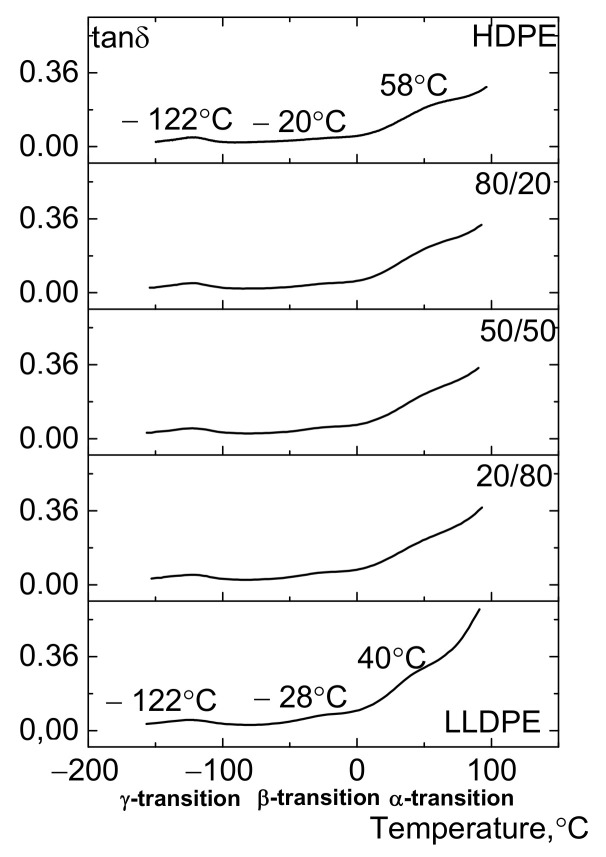
The effect of LLDPE on the loss modulus of HDPE/LLDPE blends (DMA).

Figure 14, Figure 15, Figure 16 and Figure 17 show the results of dynamic mechanical analysis of the blends. As one can see in Figure 14, the elastic modulus (rigidity and brittleness of the samples) rises with decreasing test temperature, which is caused by lower molecular mobility in the amorphous phase of polyethylene [17]. The initial HDPE and LDPE (samples 100/0 and 0/100, respectively) differ significantly in terms of their elastic modulus in the temperature range from −150 to +100 °C. The increased weight fraction of LLDPE reduces this parameter in a regular manner; the curve shape becomes more similar to that for LLDPE.

In order to interpret the low-temperature mechanical properties, we need to consider the relaxation component of strain (the loss modulus determined and tan δ by DMA). Three relaxation transitions (usually denoted as the α-, β-, and γ-transitions) are observed in the temperature range of −150 ÷ 100 °C (Figure 15 and Figure 16) [18,22,47,48]. It is believed that they are associated with mobility of the crystalline and amorphous phases of PE [49,50,51]. A hypothesis has been put forward that the α-transition is caused by the occurrence of mobility of backbone segments within the crystals before they start to melt. The transition temperature depends on crystal thickness and is independent of molecular weight and the type of side branches. The β-relaxation is associated with glass transition of the amorphous phase of PE, which is determined by changes in mobility of the branched side sections of chains [49,50,51,52]. The γ-transition is related to rotation of four segments of the carbon chain (the Shatsky/crankshaft mechanism) and was observed at −120 °C.

In this study, the low-temperature mechanical properties of polyethylene samples correspond to the β-transition lying in the temperature range of −75 ÷ 0 °C (Figure 14). Figure 14 demonstrates that adding LLDPE to HDPE shifts the β-transition peak to lower temperatures. Thus, whereas the peak for HDPE was observed at −23 °C, adding 30% LLDPE makes it shift to −28 °C, while for the initial LLDPE the peak was observed at −33 °C. The area S under the β-transition (S_β_) curve increases as LLDPE is added to HDPE due to the increasing contents of short-chain branches and the amorphous fraction, which was confirmed by the NMR data (the number of CH_3_ groups per 1000 C atoms) and the data on the content of o-xylene-soluble fraction (Table 6, Figure 15 and Figure 16).

Figure 16 shows that adding 30 wt.% of LLDPE to HDPE increases the area S under the β-transition curve 1.5-fold, and this process is accompanied by an increase in relative elongation at break at −45 °C. As we have demonstrated earlier [34], the bimodal HDPE/ter-co-LLDPE blends are thermodynamically compatible, which is the general reason why mechanical properties of these compositions produced by melt blending are improved at subzero temperatures. However, this seems to be insufficient for improving the Izod impact strength at −40 °C in the HDPE/LLDPE blend (70/30 ÷ 50/50), which is presumably caused by the reduction of molecular weight of the composition.

According to the literature data [22,47], no unambiguous interpretation of the relaxation β-transition has been given. However, it is known for sure that this transition is related to motion with respect to long chain segments. The observed relaxation phenomena are related to displacements in the interlamellar amorphous regions; therefore, they might be associated with glass transition of polyethylene [18,22].

The accurate glass-transition temperature for the amorphous region depends on the features of polyethylene structure (molecular weight, MWD, as well as type, content and distribution of comonomers and their amorphous regions, and the supramolecular structure) [18]. As demonstrated by the DSC data, the glass transition temperature of the “notionally amorphous” LLDPE fraction having lower molecular weight and being soluble in *o*-xylene is observed at −51°C. Therefore, adding LLDPE to HDPE increases the content of the “notionally amorphous” fraction XS from 1.4 to 4.1 % mass. (Figure 16) and shifts the ductile-to-brittle transition zone to lower temperatures. Hence, the temperature of −40 °C and the region below it is the “boundary zone,” where the molecular mobility of PE is significantly limited. It is also noteworthy that the temperature of −45 °C is the minimal or the threshold temperature for the operating conditions (transportation, loading/unloading, and construction/assemblage) of polyethylene-coated pipes [9]. Therefore, taking into account the aforementioned facts, we have placed special focus on testing mechanical properties of polyethylene materials at temperatures of −40 ÷ 45 °C. In our opinion, the temperature range from −40 to −60 °C is the region of interest for the researchers who strive to produce polyethylene highly resistant to subzero temperatures. Of course, the relatively new metallocene-based polyolefins characterized by ultra-low-density and high amorphization degree are even more promising materials for HDPE modification [53].

## 4. Conclusions

The investigation of the low-temperature properties of polyethylene has shown that it would be more reasonable to use such parameters as the relative elongation at break at −45 °C and the Izod impact strength at −40 °C instead of ductile-to-brittle transition temperature for assessing frost resistance because the proposed parameters are more adequate and suitable for evaluating the behavior of PE-based materials at subzero temperatures. The test temperatures of −40 ÷ 45 °C are the “threshold” temperatures for bimodal HDPE at which its frost resistance can be measured. It has been demonstrated that while the frost resistance problem is not relevant for HDPE (characterized by low MFI = 0.2) used to manufacture plastic pipes because of the high Ɛ_b_^−45°C^ and A_Izod_^−40°C^ values, modification is needed for HDPE (with MFI ≥ 1.0–2.0) used for manufacturing steel pipe coatings with a much smaller layer thickness because low-temperature mechanical properties are significantly (severalfold) deteriorated with decreasing molecular weight of the polymer (and increasing MFI).

When choosing low-density modifying agents for HDPE, it was revealed that the LLDPE terpolymer (C_2_/C_4_/C_6_) exhibits the best low-temperature properties compared to those of homo-LDPE and binary LLDPE copolymer (C_2_/C_4_) due to a combination of short-chain branching involving the 1-butene/1-hexene blend and slight modification of the MWD according to the two-reactor polymerization scheme.

It has been demonstrated that melt blending of HDPE and ter-co-LLDPE allows one to improve the relative elongation at break at −45 °C while retaining the Izod impact strength at −40 °C. The DMA data prove that 30–50% of ter-co-LLDPE added to HDPE as a modifying agent shifts the peak corresponding to the β-transition toward low temperatures and gives rise to polyethylene resins characterized by enhanced resistance to tensile strain at subzero temperatures.

Overall, it has been demonstrated that the multimodal HDPE/LLDPE blends (in terms of their density and molecular weight) can exhibit high elastic properties at temperatures below −40 °C.

The reported positive results have been confirmed by the industrial tests of the HDPE/LLDPE blend in manufacturing of coated pipes, which have demonstrated that these blends are easily processable and exhibit good performance characteristics. These blends with improved low-temperature properties are of significant interest for commercial manufacturing of both polyolefins and products based on them. In the case of external coatings for steel pipes, the rheological and low-temperature properties (tensile and impact strength) can be successfully adapted by varying the characteristics of HDPE and LLDPE, as well as their ratio in the blends.

## Figures and Tables

**Table 1:** Characteristics of high-density polyethylene samples.

Sample Name	Type of Comonomer	Comonomer Content, wt.%.	Density, g/cm^3^	MFI, g/10 min (5.0 kg/190 °C)
HDPE-1	1-Hexene	2.2	0.948	0.2
HDPE-2	1-Hexene	2.1	0.949	0.35
HDPE-3	1-Hexene	2.2	0.948	0.5
HDPE-4	1-Hexene	2.0	0.950	1.1
HDPE-5	1-Hexene	2.1	0.948	1.6
HDPE-6	1-Hexene	2.3	0.947	2.0

**Table 2:** Characteristics of the low-density polyethylene samples.

Sample Name	Type of the Comonomer	Density, g/cm^3^	MFI, g/10 min (5.0 kg/190 °C)
LDPE-1	-	0.920	6.4
LLDPE-1 (bi-co-LLDPE)	1-Butene	0.918	7.7
LLDPE-2 (ter-co-LLDPE)	1-Butene/1-Hexene	0.918	8.2

**Table 3:** The data on the weight ratio between the binary HDPE–LLDPE blends.

Name of the Composition	Weight Fraction of LLDPE, wt.%	Weight Fraction of HDPE, wt.%
100/0 (HDPE)	0	100
80/20	20	80
70/30	30	70
60/40	40	60
50/50	50	50
40/60	60	40
20/80	80	20
0/100 (LLDPE)	100	0

**Table 4:** The results of analyzing the HDPE samples 1–6.

Sample Name	Density, g/cm^3^	MFI *, g/10 min	*M*_w_, kg/mol	*M*_n_, kg/mol	*M*_z_, kg/mol	*M*_w_/ *M*_n_	Crystallinity,%	*T*_db_, °C
HDPE-1	0.948	0.2	250	13	980	19.0	67	<−70
HDPE-2	0.949	0.35	210	12	930	17.5	68	<−70
HDPE-3	0.948	0.5	180	11	820	16.0	67	<−70
HDPE-4	0.950	1.1	160	11	725	14.5	69	<−70
HDPE-5	0.948	1.6	150	13	695	11.5	67	<−70
HDPE-6	0.947	2.0	140	16	670	8.8	65	<−70

* Conditions: load, 5.0 kg; temperature, 190 °C; *T*_db_ is the ductile-to-brittle transition temperature.

**Table 5:** Characteristics of ethylene polymers having a linear and branched structure.

No.	Name	Type of PE *	ρ_+23_ **, g/cm^3^	MFI_5.0_, g/10 min	*M*_w_, kg/mol	*M*_w_/ *M*_n_	Content (Type) of Branches/ 1000 C (^13^C NMR)	*T*_melt,_ °C	Crystallinity, %	XS ***, wt.%	Ɛ_b_^+23°C^, %
1	LDPE	Homo- polymer C_2_	0.920	6.4	180	10.5	2.3 (Et), 8.3 (Bu); 3.1(Am); 3.7 ****	109	36	5.5	960
2	LLDPE-1 (bi-co-LLDPE)	Binary copolymer C_2_/C_4_	0.918	7.7	89	3.2	22.4 (Et)	125	41	9.5	930
3	LLDPE-2 (ter-co-LLDPE)	Ter-polymer C_2_/C_4_/C_6_	0.918	8.2	88	5.2	16.4 (Et)/4.5 (Bu) (Σ_Et+Bu_ = 20.9)	126	41	14.0	590

* C2—ethylene, C4—1-butene, C6—1-hexene; ** ρ +23—density; *** XS—fractions. soluble in o-xylene; ****—terminal methyl groups of the main chain and long branch chain, where Et denotes the ethyl side chains, Bu denotes the butyl side chains, and Am denotes the amylene side chains.

**Table 6:** The effect of the HDPE/LLDPE weight ratio on the MFI, molecular characteristics, and the content of CH_3_ groups and o-xylene-soluble fractions of the compositions.

No.	HDPE/ LLDPE Weight Ratio	Density, g/cm^3^	MFI 5 kg g/10 min	*M*_w_, kg/mol	*M*_z_, kg/mol	*M*_w_/ *M*_n_	CH_3_/ 1000 C *, (IR Spectroscopy Data)	*T*_m_, °C	Crystallinity, %	XS, wt.%
1	100/0	0.950	1.1	160	725	14.5	5.2	135	69	1.4
2	80/20	0.945	2.3	145	650	13.0	9.4	134	63	3.6
3	70/30	0.942	2.5	135	610	10.6	10.9	132	60	4.1
4	60/40	0.940	3.2	130	560	9.3	12	130	58	4.5
5	50/50	0.936	3.9	129	510	8.2	-	129	53	-
6	40/60	0.933	4.7	110	460	7.3	17	129	49	6.9
7	20/80	0.928	6.0	100	380	5.9	22	128	45	9.7
8	0/100	0.918	8.2	88	270	5.2	26	126	41	14.0

* The content of branches (C_2_H_5_ + C_4_H_9_ + CH_3_ terminal)/1000 C according to the IR spectroscopy data.

## Data Availability

The data presented in this study are available on request from the corresponding author.
